# Preoperative Central Sensitization Worsens Pain and Dissatisfaction Following Unicompartmental Knee Arthroplasty

**DOI:** 10.3390/medicina61050912

**Published:** 2025-05-18

**Authors:** Man-Soo Kim, Keun-Young Choi, Yong In

**Affiliations:** Department of Orthopaedic Surgery, Seoul St. Mary’s Hospital, College of Medicine, The Catholic University of Korea, 222, Banpo-daero, Seocho-gu, Seoul 06591, Republic of Korea; kms3779@naver.com (M.-S.K.); heaxagon@hanmail.net (K.-Y.C.)

**Keywords:** central sensitization, pain, dissatisfaction, unicomparmental knee arthroplasty

## Abstract

*Background and Objectives*: Central sensitization (CS) has been identified as a significant factor influencing persistent pain and dissatisfaction following total knee arthroplasty (TKA). However, its effect on unicompartmental knee arthroplasty (UKA) remains largely unexplored. Unlike TKA, UKA preserves most native knee structures, with less bone cut, leading to different postoperative pain mechanisms. Nevertheless, the revision rate for unexplained pain following UKA is higher than after TKA. This study investigates the influence of preoperative CS on pain and dissatisfaction after UKA. *Materials and Methods*: This retrospective cohort study included 121 patients who underwent primary UKA for medial compartment osteoarthritis of the knee. Patients were screened for CS preoperatively using the Central Sensitization Inventory (CSI) and categorized into a CS group (CSI ≥ 40; n = 26) and a non-CS group (CSI < 40; n = 95). Clinical outcomes, including the Western Ontario and McMaster Universities Osteoarthritis Index (WOMAC), Forgotten Joint Score (FJS), and patient satisfaction, were assessed at the 2-year postoperative follow-up visit. A multivariate regression analysis was used to determine the risk factors for postoperative dissatisfaction. *Results*: The CS group reported significantly worse postoperative WOMAC pain, function, and total scores than the non-CS group (all *p* < 0.05). FJS was also significantly worse in the CS group than in the non-CS group (64.4 vs. 72.7, respectively, *p* = 0.005). Patient satisfaction was significantly lower in the CS group than in the non-CS group (65.4% vs. 95.8%, respectively, *p* < 0.001). The multivariate logistic regression analysis demonstrated that patients with a CSI score ≥ 40 had an 11.349-fold increased likelihood of dissatisfaction after UKA (95% CI: 2.315–55.626, *p* = 0.003). *Conclusions*: This study underscores the importance of recognizing CS as a critical determinant of postoperative pain and functional recovery following UKA. Patients with high CSI scores experience greater pain, increased joint awareness, and overall poorer satisfaction despite technically successful surgeries.

## 1. Introduction

Unicompartmental knee arthroplasty (UKA) is a well-established surgical procedure for treating unicompartmental osteoarthritis (OA), offering a less invasive alternative to total knee arthroplasty (TKA) with generally favorable outcomes [1,2,3,4]. However, a certain proportion of UKA patients experience persistent postoperative pain and discomfort, even in the absence of clear radiographic abnormalities or mechanical complications [5,6,7]. Among all revision cases, the proportion of revisions performed due to unexplained pain was higher after UKA (23%) than TKA (9%), raising concerns about factors influencing postoperative pain perception [5].

Persistent postoperative pain after knee arthroplasty has traditionally been attributed to peripheral mechanisms such as residual inflammation, tissue damage, or prosthetic malalignment, which activate peripheral nociceptors and lead to peripheral sensitization [8,9]. However, recent evidence suggests that in many chronic pain cases, particularly without clear anatomical abnormalities, central sensitization (CS) may play a key role. CS is a maladaptive response of the central nervous system marked by heightened pain sensitivity, reduced inhibitory control, and glial activation, often leading to allodynia and hyperalgesia [9,10]. This central amplification of pain signals—also referred to as “centralized pain,” “central augmentation,” or “pain hypersensitivity”—can persist long after the initial surgical insult has resolved [9,10]. In such cases, the brain and spinal cord essentially ‘turn up the volume’ of pain processing, independent of ongoing peripheral input [9,10]. This phenomenon helps explain why some patients report severe pain after knee arthroplasty despite good surgical outcomes and the absence of detectable complications [9,10].

Recent research has explored the role of CS in persistent pain following joint arthroplasty [8,9,10]. CS is a condition characterized by an exaggerated pain response due to alterations in central nervous system processing [8,9,10]. It is mediated by neurotransmitters such as serotonin and norepinephrine and leads to increased sensitivity to pain stimuli, lowered pain thresholds, and prolonged pain perception even after the initial source of pain has been resolved [8,9,10]. Patients with CS often report widespread pain, hyperalgesia, and allodynia, which can significantly affect their recovery following orthopedic procedures [8,9,10].

Previous studies have established a link between CS and poor outcomes following TKA [11,12,13,14,15,16]. Patients with high preoperative pain levels and low pain thresholds are more likely than others to experience severe postoperative pain and dissatisfaction, despite successful surgical intervention [11,12,13,14,15,16]. However, the role of CS in UKA remains relatively underexplored. Given that UKA is a less invasive procedure that preserves the surrounding soft tissues, it might be assumed that patients would experience less postoperative pain than TKA patients [3]. Persistent pain in a subset of UKA patients suggests that factors beyond structural abnormalities could be contributing to suboptimal outcomes [7].

Understanding the association between CS and postoperative pain in UKA patients is crucial for improving patient outcomes. Identifying patients with preoperative CS could allow for the implementation of targeted perioperative strategies to mitigate its effects. The purpose of this study was to investigate whether preoperative CS is associated with worse postoperative outcomes, including pain, function, and satisfaction, in patients undergoing UKA. We hypothesized that patients with preoperative CS would report inferior clinical outcomes and lower satisfaction following UKA compared to those without CS.

## 2. Materials and Methods

### 2.1. Study Design

This study used a retrospective cohort design to evaluate the effects of CS on postoperative outcomes following UKA. The study was conducted at a single tertiary medical center, and all procedures were performed by a single experienced orthopedic surgeon to minimize variability in surgical technique. Ethical approval for this study was obtained from the institutional review board, and informed consent was obtained from all patients prior to participation.

### 2.2. Patient Selection

**Figure 1:**
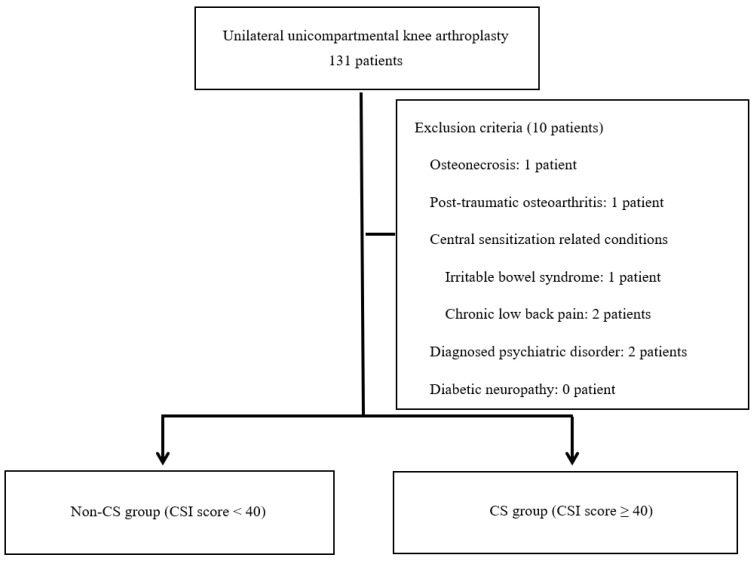
Participant flow diagram. CS: Central Sensitization, CSI: Central Sensitization Inventory.

Data from 131 patients who underwent unilateral UKA between 2014 and 2022 were initially included in the study. The inclusion criteria were as follows: (1) isolated medial compartment OA with an intact anterior cruciate ligament, (2) correctable varus deformity (less than 10 degrees), (3) minimum two-year follow-up period, and (4) no history of prior knee surgery on the affected side. Patients were excluded if they had: inflammatory arthritis (e.g., rheumatoid arthritis), osteonecrosis affecting the knee joint (1 patient), post-traumatic OA (1 patient), a history of knee infection, a documented history of CS-related conditions such as irritable bowel syndrome (1 patient) and chronic low back pain (2 patients), prior use of centrally acting agents or patients with diagnosed psychiatric conditions known to affect CS, such as anxiety or depressive disorders (2 patients), incomplete clinical outcome data (2 patients), or a subsequent operation on either knee during the follow-up period (1 patient). All patients were clinically screened for signs of peripheral neuropathy, including diabetic neuropathy, through neurologic examination and medical history review. Patients with confirmed or suspected peripheral neuropathy were excluded from the study. Notably, none of the enrolled participants exhibited clinical evidence of peripheral neuropathy. Following the application of those criteria, 10 patients were excluded from the study, resulting in a final cohort of 121 patients (Figure 1). All analyses were limited to patients who met the criteria for medial UKA, stratified by CS status. Patient demographics and baseline characteristics, including body mass index (BMI), comorbidities, and preoperative functional status, were collected through medical records and patient-reported questionnaires.

CS is influenced by both non-modifiable factors, such as female sex [17] and genetic predisposition [18], and modifiable factors, including poor sleep, psychological distress, and CS-related conditions like restless leg syndrome or chronic fatigue syndrome [19,20]. To minimize confounding, we excluded patients with major psychiatric disorders, pain catastrophizing tendencies, or clinical features of CS-related diseases through thorough medical record reviews and preoperative interviews. This approach aimed to enhance the specificity of our findings on the impact of CS [19,20].

### 2.3. Assessment of Central Sensitization

The Central Sensitization Inventory (CSI) is commonly used to assess CS and offers significant practical benefits in clinical settings [17,18]. The CSI is a validated self-reported questionnaire designed to evaluate symptoms associated with CS. Unlike quantitative sensory testing (QST), which objectively measures sensory responses to external stimuli, the CSI focuses on subjective symptom assessment [17,18].

The CSI questionnaire consists of 25 items that capture a broad spectrum of somatic and emotional symptoms frequently observed in individuals with CS [17,18]. These include headaches, fatigue, sleep disturbances, cognitive difficulties, and psychological distress, as well as heightened pain sensitivity that can interfere with daily life. The CSI specifically evaluates the presence of unrefreshing sleep, muscle stiffness and pain, anxiety attacks, bruxism, gastrointestinal disturbances (diarrhea/constipation), difficulty daily activities, light sensitivity, physical fatigue, widespread pain, urinary discomfort, poor sleep quality, concentration issues, skin problems, stress-related physical symptoms, depression, low energy, muscle tension in the neck and shoulders, jaw pain, dizziness or nausea triggered by certain smells, frequent urination, restless legs, memory impairment, childhood trauma, and pelvic pain [17,18].

Each item is rated on a 5-point Likert scale ranging from 0 (never) to 4 (always), with a total possible score of 0 to 100. According to Neblett et al. [18], a score of 40 or higher suggests the presence of CS. The CSI is easy to administer, takes less than 10 min to complete, and does not require specialized equipment. Additionally, because it incorporates non-painful and hypothetical scenarios, it avoids ethical concerns associated with other assessment methods. These advantages make the CSI a highly useful tool for evaluating the severity of CS-related symptoms. It is widely recognized as a reliable and validated measure for quantifying CS symptom severity [17,18] (Supplementary Figure S1).

### 2.4. Surgical Procedure and Postoperative Management

All UKA procedures were performed by a single surgeon using a standardized minimally invasive approach. The same cemented, mobile-bearing (MB) UKA system (Microplasty Oxford MB UKA, Zimmer Biomet, Warsaw, IN, USA) was used in all cases to ensure consistency across patients. All operations were performed under general anesthesia through the mini-medial parapatellar approach. A pneumatic tourniquet that inflated to 300 mmHg was applied. The medial meniscus was resected, and the osteophytes of the medial femoral condyle and tibial plateau were carefully removed. With the Microplasty Oxford MB UKA system, the femoral gap-sizing spoons and G-clamp were used to assess the femoral component size, tibial cutting depth, and orientation. After determining the size of the medial femoral condyle and evaluating the gap between the femur and tibia using a series of femoral sizing spoons, the most suitable spoon was selected and positioned on the MFC. After securing the gap-sizing spoon to the tibial resection guide with the G-clamp, the guide was aligned parallel to the long axis of the tibia in both the coronal and sagittal planes. The tibia was then resected, and an intramedullary rod with a distal linking feature was inserted into the femoral canal until fully seated. With the IM link engaged, the femoral drill guide was positioned on the tibial cut surface to determine the femoral component position. A posterior femoral condylar cut was performed along the femoral cutting guide. Subsequently, distal femoral milling was carefully performed to balance the flexion and extension gaps without ligament release. Cement fixation was used for all femoral and tibial components. A compressive bandage was applied postoperatively. Patients were allowed full weight-bearing on postoperative day 1 with the assistance of a walker, and a structured physical therapy program was initiated to optimize range of motion and quadriceps strengthening. The same preemptive multimodal analgesic regimen was applied to all patients. Postoperatively, intravenous patient-controlled analgesia, programmed to deliver 1 mL of a 100-mL solution containing 2000 μg of fentanyl, was used. Once the patients restarted oral intake, they received 10 mg of oxycodone every 12 h for one week, along with 200 mg of celecoxib, 37.5 mg of tramadol, and 650 mg of acetaminophen every 12 h for six weeks. Subsequent visits were scheduled at two weeks, six weeks, three months, six months, and one year, with yearly visits thereafter.

### 2.5. Outcome Measures

Postoperative outcomes were assessed at baseline and two years using validated patient-reported outcome measures (PROMs). Pain, stiffness, and function were evaluated using the Western Ontario and McMaster Universities Osteoarthritis Index (WOMAC), a well-established tool for assessing clinically relevant outcomes in patients undergoing treatment for OA of the hip or knee [19]. Additionally, joint awareness during daily activities was measured using the Forgotten Joint Score (FJS), which has been validated as a key indicator of successful joint arthroplasty [20]. To assess patient satisfaction, the new Knee Society Satisfaction (KSS) score, a five-item questionnaire designed to evaluate satisfaction with daily activities, was used. Patients were categorized as satisfied (total score 21–40) or dissatisfied (0–20), following the scoring system developed by Noble et al. [21] In addition to PROMs, postoperative complications—infection, implant loosening, and the need for revision surgery—were systematically recorded to provide a comprehensive evaluation of surgical outcomes.

## 3. Statistical Analysis

All data are presented as the mean and standard deviation. Data were compared between the non-CS group (preoperative CSI score < 40) and the CS group (preoperative CSI score ≥ 40). Continuous variables were analyzed using independent *t*-tests, and categorical variables were compared using chi-square tests. Patient demographics and surgical characteristics were collected as dependent variables to identify risk factors for patient dissatisfaction. A multivariable logistic regression analysis was conducted to evaluate associated factors, using a backward elimination approach to retain significant predictors of postoperative dissatisfaction following UKA. Odds ratios were calculated with 95% confidence intervals (CIs). Statistical analysis was performed using SPSS^®^ for Windows v21.0, with *p* < 0.05 indicating statistical significance.

## 4. Results

The final cohort comprised 121 patients who underwent UKA and met the inclusion criteria. Participants included individuals aged 42 to 82 years who met the inclusion criteria for UKA. Patients were stratified into two groups based on their preoperative CSI scores: 95 patients (78.5%) had CSI scores below 40 (non-CS group), and 26 patients (21.5%) had CSI scores of 40 or higher (CS group). The two groups did not differ significantly in demographic characteristics or surgical factors, except for CSI scores (Table 1).

Preoperative WOMAC subscores differed significantly between the groups (*p* < 0.05). Before surgery, WOMAC pain, function, and total scores were significantly worse in the CS group than in the non-CS group, indicating worse baseline symptoms (all *p* < 0.05).

In both groups, all WOMAC subscores (pain, function, and total scores) showed significant improvements postoperatively, compared with the preoperative values (all *p* < 0.05) (Table 2). However, the mean postoperative WOMAC pain score was still higher in the CS group than the non-CS group (7.8 vs. 1.8, respectively, *p* < 0.001) 2 years postoperatively. Similarly, the postoperative total WOMAC score was significantly higher in the CS group than in the non-CS group (30.5 vs. 12.8, respectively, *p* < 0.001). The improvement in preoperative to postoperative WOMAC subscores (pain, function, and total) was significantly greater in the non-CS group than in the CS group (all *p* < 0.05) (Table 2).

The FJS, which evaluates joint awareness during daily activities, was significantly lower in the CS group than in the non-CS group (64.4 vs. 72.7, respectively, *p* = 0.005). This finding suggests that patients with CS were more likely than those without CS to be aware of their knee joint postoperatively (Table 3).

The new KSS score was 33.1 in the non-CS group and 25.5 in the CS group (*p* < 0.001). Among non-CS group patients, 91 (95.8%) were satisfied with their UKAs, whereas only 17 (65.4%) patients in the CS group reported satisfaction with the surgery (*p* < 0.001). Satisfaction in the CS group was, thus, significantly lower than in the non-CS group, not only for light daily activities such as sitting, lying in bed, and getting out of bed, but also for physically demanding tasks such as household chores and recreational or leisure activities (all *p* < 0.05) (Table 4).

The multivariate logistic regression analysis demonstrated that patients with a CSI score ≥ 40 had a 6.526-fold increased likelihood of dissatisfaction after UKA (95% CI: 2.298–18.531, *p* < 0.001), compared with patients with a CSI score < 40. The associations remained statistically significant after adjusting for age, gender, BMI, American Society of Anesthesiologists grade, preoperative flexion contracture and further flexion, preoperative hip/knee/ankle angle, and preoperative WOMAC total scores (Table 5). No patient in either group experienced complications requiring additional surgery or revision during the follow-up period.

To assess whether the sample size was sufficient to detect the observed difference in satisfaction between the groups, we conducted a post-hoc power analysis using the observed satisfaction rates (95.8% in the non-CS group vs. 65.4% in the CS group). Based on a two-sided α of 0.05 and group sizes of 92 and 26, the power to detect a statistically significant difference was calculated to be 95.7% using a two-proportion z-test. This suggests that despite the unequal group sizes, the study was adequately powered to detect the observed effect.

## 5. Discussion

The findings of this study highlight the significant role that CS plays in postoperative pain and dissatisfaction following UKA. Patients with preoperative CS, identified as a CSI score of 40 or higher, demonstrated significantly worse postoperative pain scores, increased joint awareness, and inferior functional outcomes compared with those without CS. These results provide valuable insights into the influence of preoperative pain processing mechanisms on surgical recovery and patient satisfaction.

Several tools are available to assess CS, including QST and advanced imaging modalities such as functional magnetic resonance imaging (fMRI). QST objectively measures pain and sensory thresholds to external stimuli at local or remote sites, and has been widely used to detect abnormal pain processing suggestive of CS [21,22]. fMRI has also provided insights into altered brain structure and neurochemical activity in CS, such as decreased cortical thickness and elevated glutamate levels [23,24]. In this study, we chose to use the CSI, a validated self-reported questionnaire specifically designed to identify symptoms associated with CS [13,19,20,25,26]. While CSI is a self-reported measure and inherently subject to potential response bias, it offers several advantages that help mitigate these concerns. First, the CSI has been shown to have high internal consistency and reliability, and a cutoff score of 40 has been validated to identify patients with clinically significant central sensitization [19,20,25]. It is composed of items that assess not only pain but also other non-painful characteristic symptoms of CS, such as fatigue, poor sleep, and concentration difficulties, reducing the likelihood of misclassification due to isolated pain complaints [13,19,20,25,26]. Second, to minimize confounding factors, we excluded patients with known CS-related conditions (e.g., fibromyalgia, irritable bowel syndrome) and psychiatric disorders, both of which are known to influence CSI scores [19,20,25]. This allowed for a more specific assessment of CS-related symptoms within the context of knee OA [19,20,25]. Finally, although objective methods such as QST can provide valuable information about the sensory nervous system and local/distant hyperalgesia, they require specialized equipment and training, and are not always feasible in a retrospective design [19,20,25]. The CSI, in contrast, is efficient (<10 min), easy to administer, and ethically unproblematic, making it an appropriate tool for screening CS in clinical populations when used with appropriate exclusion criteria [13,19,20,25,26]. Given these benefits and considering the retrospective nature of our study, CSI provided an efficient and robust tool to stratify patients based on their CS status [19,20,25].

Patients with preoperative CS experienced significantly worse postoperative pain and functional outcomes than those without CS, despite the theoretical advantages of UKA over TKA, such as a smaller incision, preservation of most soft tissues, and less bone resection [3]. Our findings reveal that the CS group reported notably higher pain scores two years postoperatively. No previous studies investigated the effects of preoperative CS on UKA outcomes. However, numerous studies have examined the relationship between preoperative CS and clinical outcomes following TKA [11,12,13,14,15,16,27,28], and the evidence suggests that patients with preoperative CS are at greater risk than those without CS for chronic postoperative pain [11,12,13,14,15,16,27,28]. Martinez et al. [28] found that TKA patients with heat hyperalgesia reported greater pain both before and after surgery and required higher doses of postoperative morphine. Similarly, Lundblad et al. [27] followed 69 patients for 18 months after TKA and noted that persistent pain was more prevalent among those with high preoperative pain levels and a lower pain threshold—both indicative of CS-related mechanisms. In another study, Kim et al. [13] reported that patients with a high CSI score (≥40) experienced more intense postoperative pain and required greater analgesic use during the first three months postoperatively. Their study also demonstrated that higher CSI scores were associated with more severe preoperative pain, persistent postoperative pain, and lower satisfaction with pain relief three months after surgery. Interestingly, although UKA is designed to preserve native ligaments and provide more natural knee kinematics, patients with CS did not appear to benefit from those advantages [3]. Instead, they continued to experience significant pain and functional limitations. This suggests that in CS patients, abnormalities in pain processing play a more important role in postoperative recovery than the specific surgical technique used. These findings further support the hypothesis that postoperative pain perception is not solely dictated by structural changes but is also influenced by alterations in central nervous system pain processing.

Preoperative CS not only contributes to persistent pain following surgery but also significantly affects functional outcomes and overall patient satisfaction. In our study, the CS group exhibited poorer WOMAC function and total scores than the non-CS group at the two-year follow-up visit. Additionally, the FJS scores were notably lower in the CS group, indicating a greater level of functional impairment. These findings align with previous studies of TKA [12,14]. Kim et al. [12] reported that the CS group showed significantly inferior preoperative and postoperative WOMAC function and total scores than the non-CS group. Similarly, Koh et al. [14] found that the CS group experienced worse quality of life and greater functional disability than the non-CS group after TKA. In addition, one of the most striking findings of our study is the significant effect of CS on patient satisfaction following UKA. Whereas 88% of patients in the non-CS group reported being satisfied with their surgical outcomes, only 62% of those in the CS group expressed satisfaction (*p* < 0.01). Sasaki et al. [16] demonstrated that preoperative CS was also negatively associated with postoperative EQ-5D scores in TKA patients. Moreover, Koh et al. [14], using the same new KSS score as in our study, showed that patients in the CS group were significantly more dissatisfied than those in the non-CS group. Further supporting this, our multivariate regression analysis identified CS as a significant predictor of dissatisfaction. The preoperative CSI score (adjust odds ratio = 11.349, *p* = 0.003) was independently associated with lower satisfaction rates. These findings underscore the importance of preoperative CS assessment, which may help screen high-risk patients and enable tailored interventions, including education and preemptive medication, to improve postoperative satisfaction.

CS is strongly associated with poor clinical outcomes following UKA for several reasons. First, patients with CS often have higher preoperative expectations than those without CS. Specifically, they anticipate greater pain relief and psychological well-being after surgery [29]. Although high expectations can sometimes contribute to favorable postoperative outcomes [30], excessively high expectations are closely linked to dissatisfaction and poor clinical results [31]. Second, the heightened pain sensitivity of CS patients negatively affects their postoperative outcomes [29]. Individuals with CS experience increased pain perception, often presenting with hyperalgesia and allodynia as characteristic symptoms [9]. This heightened sensitivity can play a crucial role in difficulties with post-surgical pain and recovery, further contributing to suboptimal clinical results [29]. Third, CS patients tend to have higher minimal clinically important difference (MCID) thresholds than non-CS patients. As a result, their overall postoperative outcomes tend to be worse, and the likelihood of achieving the MCID is significantly lower [12]. For those reasons, patients with CS are more prone than those without CS to experience persistent pain and inferior outcomes following UKA.

Effective management of CS, particularly in patients undergoing knee arthroplasty, requires a multidimensional approach that extends beyond conventional surgical intervention [14,32,33,34]. First, preoperative education and expectation setting play a crucial role. Numerous studies have demonstrated that preoperative expectations are closely linked to postoperative satisfaction and clinical outcomes [33,34]. For patients with CS, it is especially important to provide detailed explanations about the potential impact of CS on pain perception and recovery trajectory [33,34]. By aligning patient expectations with realistic outcomes, clinicians can mitigate dissatisfaction and enhance shared decision-making [33,34]. Furthermore, specific education on the nature of CS and its role in persistent postoperative pain should be incorporated into preoperative counseling sessions for patients with knee OA scheduled for UKA [33,34]. Second, pharmacologic treatment targeting central pain mechanisms can be an effective adjunct. Among the available agents, the serotonin-norepinephrine reuptake inhibitor (SNRI) duloxetine has shown particular promise in CS-associated pain [32]. In a randomized controlled trial by Koh et al., patients with CS undergoing TKA who received duloxetine experienced not only significant pain reduction starting two weeks postoperatively, but also notable improvements in mood, mental health, sleep quality, and social functioning [14]. These findings suggest that targeting descending pain modulation pathways may alleviate both sensory and affective components of CS-related pain [14]. Taken together, these strategies—preoperative patient-centered education and targeted pharmacotherapy—represent critical components in the perioperative management of patients with CS to improve both subjective and objective surgical outcomes [14,32,33,34]. Although UKA is widely regarded as a minimally invasive and function-preserving procedure, patients with CS may not fully experience its expected clinical benefits due to persistent central pain mechanisms [14,32,33,34]. This does not imply that UKA is counterproductive in CS patients, but rather highlights the need for realistic expectation-setting, individualized pain management, and possibly multimodal interventions to support postoperative recovery in this subgroup.

This study has several strengths, including a well-defined patient cohort, a standardized surgical technique performed by a single surgeon, and the use of validated outcome measures such as the WOMAC and FJS. These factors minimize variability and enhance the reliability of our findings. However, there are also limitations to consider. First, most of the patients who underwent UKAs were female (108 of 131, 89%). Although this demographic trend is well-documented in the Korean population, the underlying reasons remain unclear [35,36,37,38,39,40,41]. Second, although the data were collected prospectively, this study was conducted as a retrospective review using a single-institution database. As a result, inherent limitations such as selection bias might have influenced the findings. Third, various tools exist for assessing patient satisfaction after surgery [42]. In this study, we used the new KSS system, a validated tool designed to minimize the evaluation burden [43]. Although the KSS is widely accepted, incorporating additional assessment methods could provide a more comprehensive evaluation of patient satisfaction. Fourth, the follow-up period was limited to two years, and patient satisfaction was assessed only at the two-year postoperative mark. Longer-term studies are needed to better understand how postoperative satisfaction and its relationship with CS evolve over time. Fifth, the study might be underpowered, increasing the risk of type II errors and potentially limiting the ability to detect all relevant associations. Larger prospective studies with a broader and more diverse patient population are needed to strengthen these findings. Sixth, we used the CSI as the primary tool for assessing CS. Although the CSI is a validated and widely used screening measure [19,20], it is based on self-reported data and might not fully capture the neurophysiological aspects of CS. Future studies incorporating QST or functional neuroimaging could provide a more comprehensive understanding of CS in patients undergoing UKA [44]. Seventh, all surgeries were performed at a single institution by a single surgeon, which might limit the generalizability of the findings to other surgical settings. A multicenter study would help validate these results across different patient populations. Eighth, although we aimed to isolate the impact of CS on UKA outcomes by excluding patients with major psychiatric disorders and CS-related conditions through predefined exclusion criteria [19,20], it is important to acknowledge that completely eliminating all potential CS-associated factors is inherently challenging. Subclinical symptoms or undiagnosed comorbidities, such as mild sleep disturbances or psychological stress, may have persisted and influenced outcomes, representing a limitation of this study. Additionally, although patients with peripheral neuropathy were excluded based on clinical screening, we did not conduct formal neurologic or electrophysiological evaluations. Future studies may benefit from incorporating objective neuropathy assessments and stratifying patients by metabolic comorbidities to better isolate the effects of CS [45]. Lastly, anxiety and depression were excluded based on preoperative evaluation; we did not assess subclinical levels of anxiety or depressive symptoms postoperatively. Considering the established relationship between CS and mood disturbances, this remains a relevant limitation that may have influenced subjective outcome measures [14]. Despite those limitations, this study provides valuable insights into the relationship between CS and both pain and dissatisfaction following UKA.

## 6. Conclusions

This study underscores the importance of recognizing CS as a critical determinant of postoperative pain and dissatisfaction following UKA. Patients with high CSI scores experience greater pain, increased joint awareness, and overall poorer outcomes despite technically successful surgeries. Patients with CS should be closely monitored postoperatively and provided with appropriate pain management strategies to optimize their surgical outcomes. Future research should focus on refining these strategies and exploring innovative approaches to pain modulation in this patient population.

## Figures and Tables

**Table 1:** Comparison of demographic characteristics and surgical factors in the central sensitization and non–central sensitization groups.

Variables	Non-CS Group (CSI < 40) (n = 95)	CS Group (CSI ≥ 40) (n = 26)	Cohen’s d Effect Size	*p*-Value
Demographics				
Age (years)	61.5 ± 5.9	60.2 ± 7.4	0.21	0.366
Gender (Female, %)	84 (88.4%)	24 (22.2%)		0.732
BMI (kg/m^2^)	25.6 ± 3.0	26.7 ± 3.3	−0.36	0.114
CSI score	22.7 ± 9.8	45.2 ± 4.2	−2.52	<0.001
ASA grade				
1	6 (6.3%)	2 (7.7%)		0.681
2	89 (93.7%)	24 (92.3%)		
Operation side (Right, %)	41 (43.2)	14 (53.8%)		0.514
Specific comorbidities				
Hypertension	29 (30.5%)	7 (19.4%)		0.812
Diabetes	16 (16.8%)	3 (11.5%)		0.762
Cardiac disease	12 (12.6%)	4 (15.4%)		0.746
Cerebrovascular event	5 (5.3%)	0 (0%)		0.584
Kidney disease	2 (2.1%)	0 (0%)		0.615
Thyroid disease	5 (5.3%)	1 (3.8%)		0.618
Pulmonary disease	10 (10.5%)	1 (3.8%)		0.453
Liver disease	3 (3.2%)	0 (0%)		0.481
Preoperative FTA (°)	Valgus 5.5 ± 2.7	Valgus 5.5 ± 3.2	0.0	0.970
Preoperative HKA (°)	Varus 0.6 ± 3.4	Varus 1.2 ± 3.8	−0.17	0.441
Postoperative FTA (°)	Valgus 1.8 ± 3.2	Valgus 2.3 ± 2.6	−0.16	0.454
Postoperative HKA (°)	Varus 4.7 ± 3.4	Varus 4.4 ± 3.2	0.09	0.664
Preoperative FC	3.4 ± 5.4	3.1 ± 5.3	0.06	0.774
Preoperative FF	127.7 ± 11.7	126.2 ± 15.4	0.12	0.583
Postoperative FC	0.3 ± 1.2	0.8 ± 2.7	−0.31	0.209
Postoperative FF	129.7 ± 3.8	129.0 ± 3.0	0.19	0.361

Values are presented as means and standard deviations or n (%). BMI, Body Mass Index; CSI, Central Sensitization Inventory; ASA, American Society of Anesthesiologists; FTA, Femorotibial Angle; HKA, Hip/Knee/Ankle Angle; FC, Flexion Contracture; FF, Further Flexion.

**Table 2:** Preoperative and postoperative patient-reported outcomes.

Variables	Non-CS Group (CSI < 40) (n = 95)	CS Group (CSI ≥ 40) (n = 26)	Cohen’s d Effect Size	*p*-Value
Preoperative				
WOMAC Total	54.4 ± 11.6	63.7 ± 12.0	−0.80	0.001
WOMAC Pain	14.5 ± 4.2	17.5 ± 4.1	−0.72	0.001
WOMAC Stiffness	4.4 ± 2.9	4.8 ± 2.0	−0.15	0.509
WOMAC Function	36.1 ± 9.6	41.8 ± 8.2	−0.61	0.006
Postoperative				
WOMAC Total	12.8 ± 7.1	30.5 ± 12.0	−2.11	<0.001
WOMAC Pain	1.8 ± 1.4	7.8 ± 4.1	−2.67	<0.001
WOMAC Stiffness	1.2 ± 1.3	1.8 ± 1.7	−0.43	0.030
WOMAC Function	9.6 ± 5.4	20.8 ± 7.7	−1.88	<0.001
WOMAC Change				
WOMAC Total	41.6 ± 12.7	33.1 ± 16.7	0.62	0.006
WOMAC Pain	12.7 ± 4.5	9.7 ± 5.6	0.63	0.005
WOMAC Stiffness	3.2 ± 2.7	3.0 ± 2.2	0.08	0.632
WOMAC Function	26.5 ± 10.3	21.0 ± 10.8	0.53	0.019

Values are presented as means and standard deviations. CS, Central Sensitization; CSI, Central Sensitization Inventory; WOMAC, Western Ontario and McMaster Universities Osteoarthritis Index.

**Table 3:** Postoperative Forgotten Joint Score (FJS) results.

Variables	Non-CS Group (CSI < 40) (n = 95)	CS Group (CSI ≥ 40) (n = 26)	Cohen’s d Effect Size	*p*-Value
FJS conditions	72.7 ± 12.1	64.4 ± 16.3	0.63	0.005
1. In bed at night	1.1 ± 0.7	1.6 ± 0.9	−0.67	0.004
2. When sitting on a chair for more than 1 h	1.0 ± 0.8	1.5 ± 0.8	−0.63	0.006
3. When walking for more than 15 min	1.1 ± 0.8	1.5 ± 0.8	−0.50	0.187
4. When taking a bath/shower	1.2 ± 0.8	1.6 ± 1.1	−0.46	0.054
5. When traveling in a car	1.0 ± 0.7	1.2 ± 1.0	−0.26	0.166
6. When climbing stairs	1.1 ± 0.9	1.2 ± 1.0	−0.11	0.576
7. When walking on uneven ground	1.4 ± 0.9	1.5 ± 0.8	−0.11	0.541
8. When standing up from a low-sitting position	1.2 ± 0.8	1.6 ± 1.1	−0.46	0.042
9. When standing for long periods of time	0.8 ± 0.8	1.2 ± 1.0	−0.47	0.051
10. When doing housework or gardening	1.0 ± 0.8	1.5 ± 1.0	−0.59	0.003
11. When taking a walk or hiking	1.1 ± 0.9	1.5 ± 0.7	−0.47	0.087
12. When doing a favorite sport	0.9 ± 0.6	1.3 ± 0.8	−0.62	0.007

Values are presented as means and standard deviations. CS, Central Sensitization; CSI, Central Sensitization Inventory; FJS, Forgotten Joint Score.

**Table 4:** Comparison of satisfaction scores and proportions.

Variables	Non-CS Group (CSI < 40) (n = 95)	CS Group (CSI ≥ 40) (n = 26)	Cohen’s d Effect Size	*p*-Value
Satisfaction score				
Sitting	7.2 ± 1.3	6.3 ± 1.9	0.62	0.004
Lying in bed	7.3 ± 1.2	6.4 ± 1.7	0.68	0.002
Getting out of bed	6.8 ± 1.5	5.8 ± 2.0	0.62	0.013
Light household duties	6.2 ± 1.6	5.4 ± 1.8	0.49	0.027
Leisure recreational activities	5.5 ± 1.8	3.5 ± 1.7	1.12	<0.001
Total	33.1 ± 6.1	25.5 ± 8.8	1.12	<0.001
Proportion				
Satisfied	91 (95.8%)	17 (65.4%)		<0.001
Dissatisfied	4 (4.2%)	9 (34.6%)		

Values are presented as means and standard deviations or n (%). CS, Central Sensitization; CSI, Central Sensitization Inventory.

**Table 5:** Results of multivariate analysis of risk factors predicting dissatisfaction.

Preoperative Variables	Univariate Regression Coefficient	95% CI	*p*-Value	Multivariate Regression Coefficient †	95% CI	*p*-Value
Age	1.008	0.945–1.075	0.816	0.982	0.912–1.057	0.620
Sex (male vs. female)	1.282	0.330–4.981	0.720	0.989	0.212–4.626	0.989
BMI (kg/m^2^)	0.955	0.840–1.086	0.483	0.976	0.842–1.131	0.747
Preoperative HKA (°)	1.072	0.957–1.201	0.232	1.114	0.982–1.265	0.094
ASA grade (grade 2 vs. 1)	1.134	0.217–5.922	0.881	1.308	0.201–8.532	0.779
Preoperative FC (°)	1.016	0.939–1.099	0.691	1.018	0.921–1.126	0.722
Preoperative FF (°)	0.998	0.966–1.031	0.905	0.997	0.958–1.039	0.899
Preoperative WOMAC total score	0.977	0.944–1.012	0.192	0.999	0.960–1.039	0.960
CSI score (≥40)	5.833	2.297–14.814	<0.001	6.526	2.298–18.531	<0.001

BMI, Body Mass Index; HKA, Hip/Knee/Ankle angle; ASA, American Society of Anestheologists; FC, Flexion Contracture; FF, Further Flexion; WOMAC, Western Ontario and McMaster Universities Osteoarthritis Index; CSI, Central Sensitization Inventory. † Multivariate regression coefficient analyses were adjusted for age, gender, BMI, American Society of Anesthesiologists grade, preoperative flexion contracture and further flexion, preoperative hip/knee/ankle angle, and preoperative WOMAC total scores.

## Data Availability

The data published in this research are available on request from the corresponding author (Y.I.).

## Supplementary Materials

The following supporting information can be downloaded at: https://www.mdpi.com/article/10.3390/medicina61050912/s1, Figure S1: Central Sensitization Inventory.

## Abbreviations

CS	Central Sensitization
UKA	Unicompartmental Knee Arthroplasty
TKA	Total Knee Arthroplasty
CSI	Central Sensitization Inventory
WOMAC	Western Ontario and McMaster Universities Osteoarthritis Index
FJS	Forgotten Joint Score
OA	Osteoarthritis
CNS	Central Nervous System
QST	Quantitative Sensory Testing
MB	Mobile-Bearing
PROMs	Patient-Reported Outcome Measures
KSS	Knee Society Satisfaction
