# Supplementary material for: Preoperative Central Sensitization Worsens Pain and Dissatisfaction Following Unicompartmental Knee Arthroplasty
Source: Medicina (Kaunas). 2025 May 18;61(5):912. doi: 10.3390/medicina61050912 (PMC12113206; doi:10.3390/medicina61050912)
Supplement: Supplementary file 1 [file medicina-61-00912-s001.zip › medicina-3610039-supplementary.pdf]

1. I feel tired and unrefreshed when I wake from sleeping.	Never	Rarely	Sometimes	Often	Always
2. My muscles feel stiff and achy.	Never	Rarely	Sometimes	Often	Always
3. I have anxiety attacks.	Never	Rarely	Sometimes	Often	Always
4. I grind or clench my teeth.	Never	Rarely	Sometimes	Often	Always
5. I have problems with diarrhea and/or constipation.	Never	Rarely	Sometimes	Often	Always
6. I need help in performing my daily activities.	Never	Rarely	Sometimes	Often	Always
7. I am sensitive to bright lights.	Never	Rarely	Sometimes	Often	Always
8. I get tired very easily when I am physically active.	Never	Rarely	Sometimes	Often	Always
9. I feel pain all over my body.	Never	Rarely	Sometimes	Often	Always
10. I have headaches.	Never	Rarely	Sometimes	Often	Always
11. I feel discomfort in my bladder and/or burning when I urinate.	Never	Rarely	Sometimes	Often	Always
12. I do not sleep well.	Never	Rarely	Sometimes	Often	Always
13. I have difficulty concentrating.	Never	Rarely	Sometimes	Often	Always
14. I have skin problems such as dryness, itchiness, or rashes.	Never	Rarely	Sometimes	Often	Always
15. Stress makes my physical symptoms get worse.	Never	Rarely	Sometimes	Often	Always
16. I feel sad or depressed.	Never	Rarely	Sometimes	Often	Always
17. I have low energy.	Never	Rarely	Sometimes	Often	Always
18. I have muscle tension in my neck and shoulders.	Never	Rarely	Sometimes	Often	Always
19. I have pain in my jaw.	Never	Rarely	Sometimes	Often	Always
20. Certain smells, such as perfumes, make me feel dizzy and nauseated.	Never	Rarely	Sometimes	Often	Always
21. I have to urinate frequently.	Never	Rarely	Sometimes	Often	Always
22. My legs feel uncomfortable and restless when I am trying to go to sleep at night.	Never	Rarely	Sometimes	Often	Always
23. I have difficulty remembering things.	Never	Rarely	Sometimes	Often	Always
24. I suffered trauma as a child.	Never	Rarely	Sometimes	Often	Always
25. I have pain in my pelvic area.	Never	Rarely	Sometimes	Often	Always

Supplementary Figure S1. Central Sensitization Inventory
